# Identification of potential crucial genes and mechanisms associated with metabolically unhealthy obesity based on the gene expression profile

**DOI:** 10.3389/fgene.2025.1540721

**Published:** 2025-05-01

**Authors:** Qingqing Wang, Silu Wang, Zhanyu Zhuang, Xueting Wu, Hongkun Gao, Tianyi Zhang, Guorong Zou, Xing Ge, Yapeng Liu

**Affiliations:** ^1^ Department of Nephrology, Xuzhou Children’s Hospital, Xuzhou, Jiangsu, China; ^2^ School of Public Health, Xuzhou Medical University, Xuzhou, Jiangsu, China; ^3^ Department of Clinical Medicine, Xuzhou Medical University, Xuzhou, Jiangsu, China; ^4^ National Demonstration Center for Experimental Basic Medical Science Education (Xuzhou Medical University), Xuzhou, Jiangsu, China; ^5^ Yunlong District Center for Disease Control and Prevention, Xuzhou, Jiangsu, China

**Keywords:** metabolically unhealthy obesity, metabolic syndrome, differentially expressed genes, bioinformatics analysis, differentially expressed microRNAs

## Abstract

**Background:**

Obesity is an epidemic and systemic metabolic disease that seriously endangers human health. This study aimed to understand the transcriptomic characteristics of the blood of metabolically unhealthy obesity (MUO) and provide insight into the target genes of differently expressed microRNAs in the occurrence and development of MUO.

**Methods:**

The GSE146869, GSE145412, GSE23561, and GSE169290 datasets were analyzed to understand the transcriptome characteristics of the blood of MUO and provide insights into the target genes of differently expressed microRNAs (DEMs) in MUO. Functional and pathway enrichment analyses and gene interaction network analyses were performed to profile the function of differentially expressed genes (DEGs). In addition, miRNet 2.0, TransmiR v2.0, RNA22, TargetScan 7.2, miRDB, and miRWalk databases were used to predict the target genes of effective microRNAs.

**Results:**

A total of 189 co-DEGs were identified in at least two datasets. The 156 co-upregulated genes were enriched into 29 biological process (BP) terms and 12 KEGG pathways. Among the 29 BP terms, the immune- and metabolism-related BP terms were enriched. The 33 co-downregulated genes were enriched into two BP terms, including apoptotic process and regulation of the apoptotic process, with no KEGG pathway. The hub genes *EGF*, *STAT3*, *IL1B*, *PF4*, *SELP*, and *ITGA2B* in the gene interaction network might play important roles in abnormal BP in MUO. Among the 19 DEMs identified in the blood of the MUO group by the GSE169290 dataset, 18 microRNAs targeted 85 genes as risk factors in MUO.

**Conclusion:**

A network consisting of 18 microRNAs and 85 target genes might serve as a risk factor for metabolically unhealthy obesity.

## Introduction

Obesity is an epidemic and systemic metabolic disease that seriously endangers human health (Collaboration, 2016). Obesity is frequently associated with the abnormalities of metabolic syndrome (MS) and an increased risk of its associated conditions, such as type-2 diabetes (Regufe et al., 2020) and cardiovascular diseases (Gargiulo et al., 2020). According to metabolic indicators, obesity can be classified as “metabolically healthy obesity” (MHO) and “metabolically unhealthy obesity” (MUO) (Iacobini et al., 2019a). MHO (approximately 30% of obesity) is characterized by obesity without obesity-related metabolic abnormalities, such as hyperglycemia, hypertension, dyslipidemia, and decreased insulin sensitivity (Bervoets and Massa, 2016). Some obese populations develop MHO and are resistant to obesity-associated metabolic diseases for some time, whereas others readily develop MUO (Dagpo et al., 2020). Although the exact mechanisms remain unclear, they are believed to involve adipose tissue dysfunction, chronic inflammation, mitochondrial oxidative dysfunction, genetics, and gut microbiota, which collectively contribute to MUO pathogenesis (Longo et al., 2019; Iacobini et al., 2019b). The risk of cardiovascular and metabolic complications in the MUO population is significantly higher than in the MHO population (Okamura et al., 2018). Therefore, the prevention and reversal of the transition from MHO to MUO should be considered therapeutic goals. Thus, understanding the transcriptome characteristics and molecular determinants of the occurrence and development of MUO becomes critical, with the potential to lead to precision medicine approaches.

**TABLE 1:** Details of datasets.

Series	Public year	Groups	Samples	Tissue	Molecule
GSE146869	2020	Metabolically unhealthy obesity “metabolically healthy obesity”	14 13	Whole-blood	mRNA
GSE145412	2020	Metabolically unhealthy obesity “metabolically healthy obesity” healthy control	8 8 16	Whole-blood	mRNA
GSE23561	2010	Metabolic syndrome control	6 9	Whole-blood	mRNA
GSE169290	2021	Metabolically unhealthy obesity “metabolically healthy obesity”	16 16	Whole-blood	microRNA

High-throughput sequencing and bioinformatics provide new ideas for studying molecular mechanisms and therapeutic targets of diseases (Pareek et al., 2011). For example, in whole human blood RNA sequencing studies, the blood transcriptome in MUO is analyzed by bioinformatics methods (Plaza-Florido et al., 2021; Paczkowska-Abdulsalam et al., 2020; Grayson et al., 2011). However, all of these studies have small sample sizes. Therefore, integrating data from these studies increases the accuracy of the results.

MicroRNAs, small RNA molecules, are reported to be associated with MUO because of their role in regulating gene expressions by binding to target mRNAs (Rovira-Llopis et al., 2021). Therefore, microRNAs and their target genes are intensely studied as candidates for diagnostic and prognostic biomarkers, predictors of drug response, and therapeutic agents of diseases, including metabolic disorders (Ridolfi and Abdel-Haq, 2017). The target genes of differentially expressed microRNAs (DEMs) in neurodegenerative diseases can be predicted by several databases, such as miRNet 2.0 (Chang et al., 2020), TransmiR v2.0 (Agarwal et al., 2015), RNA22 (Miranda et al., 2006), TargetScan 7.2 (Agarwal et al., 2015), miRDB (Chen and Wang, 2020; Liu and Wang, 2019), and miRWalk (Wang et al., 2021). The combination of multiple prediction databases and RNA sequencing results can more accurately predict the target genes of microRNAs.

MicroRNAs are pivotal regulators linking obesity, inflammation, and metabolism. In adipose tissue (AT), microRNAs secreted by adipocytes, macrophages, and T cells modulate immune cell crosstalk and metabolic organ communication. Circulating microRNAs, derived from AT-resident cells or immune cells such as M1 macrophages and T cells, play dual roles: promoting or inhibiting inflammation and insulin resistance. For example, miR-155 and miR-34a enhance pro-inflammatory pathways (targeting SOCS1 and KLF4), while miR-223 and miR-146b suppress macrophage activation. These microRNAs also regulate adipocyte differentiation (e.g., miR-16-5p and miR-326) and metabolic signaling (e.g., miR-214 targeting DPP4). Circulating microRNAs, stable in body fluids, serve as promising biomarkers for obesity-related disorders (e.g., miR-27a and miR-130b correlate with obesity severity). Additionally, they offer therapeutic potential; mimics or antagomirs targeting miR-155, miR-34a, or miR-223 could attenuate AT inflammation and metabolic dysfunction (Rakib et al., 2022). Understanding microRNAs-mediated pathways in circulating cells provides insights into obesity pathogenesis and novel intervention strategies.

In the present study, GSE146869, GSE145412, GSE23561, and GSE169290 datasets were analyzed to understand the transcriptome characteristics of the blood of MUO and provide insights into the target genes of differently expressed microRNAs in MUO.

## Materials and methods

### Dataset

The data discussed in this publication were deposited in NCBI’s Gene Expression Omnibus and are accessible through GEO Series accession numbers GSE146869, GSE145412, GSE23561, and GSE169290. The GSE146869 dataset, including 14 MUO patients and 13 MHO individuals, was a gene expression dataset in human whole blood (Table 1) [9]. The GSE145412 dataset was a gene expression dataset in human whole blood of 8 MUO patients, 8 MHO individuals, 16 patients with metabolic syndrome, and 16 healthy control individuals [10]. The GSE23561 dataset was a gene expression dataset in the peripheral blood of six MS patients and nine control individuals [11]. Finally, the GSE169290 dataset was a microRNA expression dataset from the whole blood of 10 MUO patients and 10 MHO individuals [12]. In the present study, a multi-dataset strategy was employed, which led to a substantial expansion of the sample pool compared to previous investigations with limited sample sizes. By analyzing each dataset independently for differentially expressed genes (DEGs) and subsequently identifying co-DEGs present in at least two datasets, our approach effectively mitigated the influence of dataset-specific biases. This filtering mechanism ensured that only the genes whose differential expression was robust and consistent across multiple datasets were retained, thereby significantly enhancing the accuracy of our findings.

### Differentially expressed gene and microRNA analyses

The limma package of R language was applied to identify the DEGs and differential expressed microRNAs. The required data were mRNA and microRNA with |FC| > 2 and *p*-value <0.05. The volcano plot and heatmap were plotted using ggplot2 and pheatmap packages.

### Functional and pathway enrichment analyses

Both GO and KEGG pathways, which were mainly used to study DNA- and protein-related issues, are biological sequence analysis methods that can effectively cluster functional genes into different biological processes. Next, the DAVID database (https://david.ncifcrf.gov/tools.jsp) was used to perform GO and KEGG analyses on differential expression genes. These analyses were mapped with bioinformatics. The *p*-value and false discovery rate (FDR) were controlled at the 0.05 threshold.

### Gene interaction network analysis

The gene interaction networks were analyzed using the STRING database version 11.0 (http://string-db.org). In addition, the interaction networks of differential expression genes were visualized using Cytoscape 3.6.0 software.

### Prediction of target genes of differentially expressed microRNAs

miRNet 2.0 [14], TransmiR v2.0 [15], RNA22[16], TargetScan 7.2 [15], miRDB [17, 18], and miRWalk databases [19] were used to predict the target mRNAs of effective microRNAs in mammals.

## Statistics

R 3.5.1 was used for statistical analysis. P values less than 0.05 were considered statistically significant.

## Results

### Differentially expressed genes in MUO

**FIGURE 1:**
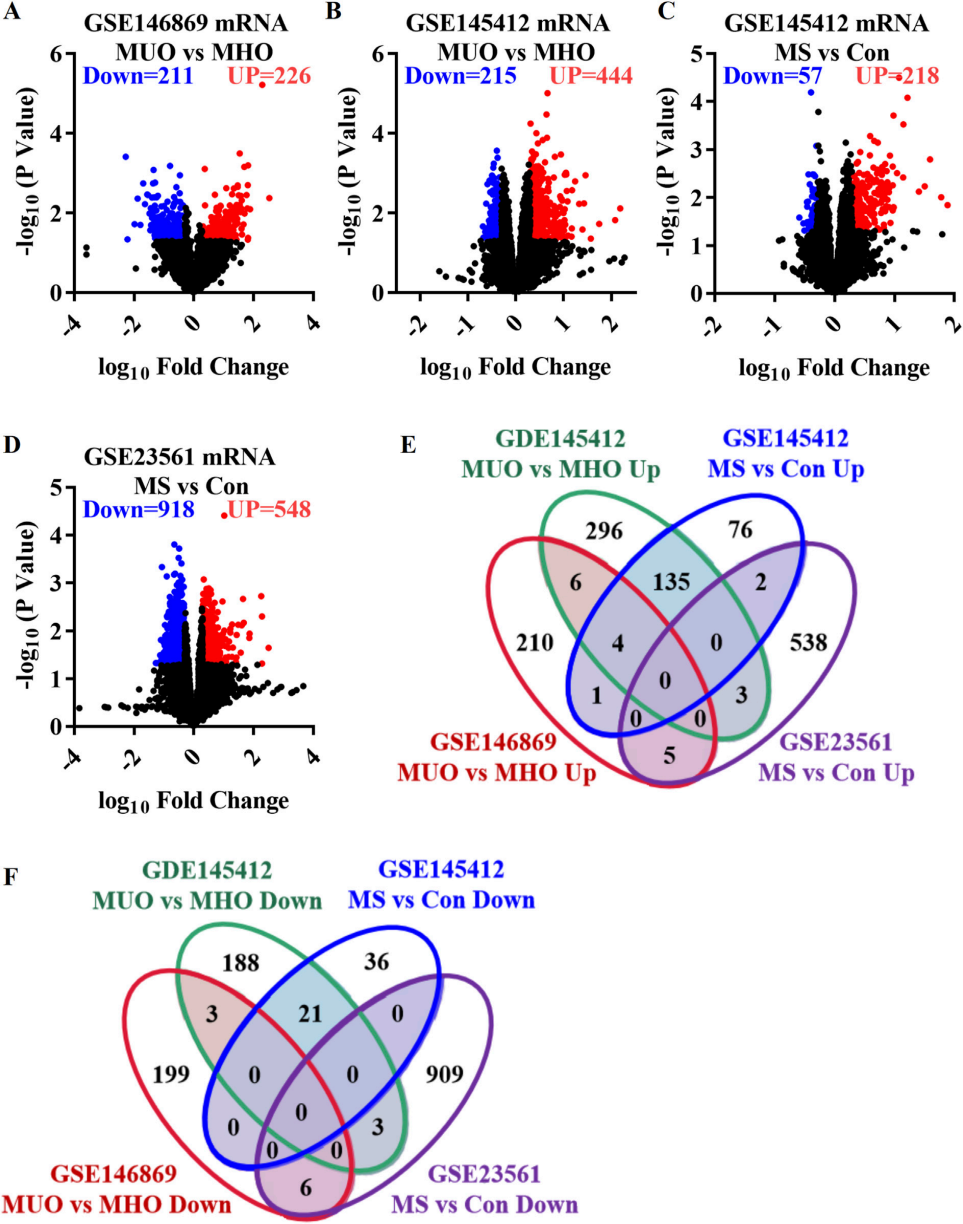
Differentially expressed genes in metabolically unhealthy obesity. **(A)** Volcano plot comparing the MUO group with the MHO group, based on the GSE146869 dataset. **(B)** Volcano plot comparing the MUO group with the MHO group, based on the GDE145412 dataset. **(C)** Volcano plot comparing the MS (metabolic syndrome) group with the control (Con) group, based on the GSE145412 dataset. **(D)** Volcano plot comparing the MS group with the Con group, according to the GSE23561 dataset. **(E)** Venn diagram presenting the upregulated genes. **(F)** Venn diagram of the downregulated genes. MS, metabolic syndrome. MUO, metabolically unhealthy obesity. MHO, metabolically healthy and obese. DEGs, differentially expressed genes.

GSE146869, GSE145412, and GSE23561 datasets were analyzed to explore the transcriptome characteristics of MUO. The GSE146869 dataset included two groups of samples: 14 MUO patients and 13 MHO individuals. Compared with the MHO group, 437 DEGs were identified in the blood of the MUO group, including 226 upregulated genes and 211 downregulated genes (Figure 1A). In the GSE145412 dataset, 8 MUO patients, 8 MHO individuals, 16 patients with metabolic syndrome, and 16 healthy control individuals were included. A total of 659 DEGs were identified in the MUO group compared with the MHO group, including 444 upregulated genes and 215 downregulated genes (Figure 1B). A total of 275 DEGs were identified in the MS group compared with the control group, including 218 upregulated genes and 57 downregulated genes (Figure 1C). The GSE23561 dataset included two groups of samples: six MS patients and nine control individuals. Compared with the control group, 1,466 DEGs were identified in the blood of the MS group, including 548 upregulated genes and 918 downregulated genes (Figure 1D). A total of 156 co-upregulated genes were identified, at least in two datasets (Figure 1E). A total of 33 co-downregulated genes were identified, at least in two datasets (Figure 1F). A total of 189 co-DEGs were found to represent the effect of metabolically unhealthy obesity. Thus, functional and pathway enrichment analyses and gene interaction network analyses on these 189 co-DEGs were further performed to profile the abnormal function and potential molecular mechanism in the occurrence and development of MUO.

### Functional and pathway enrichment analyses of the co-DEGs of MUO

**FIGURE 2:**
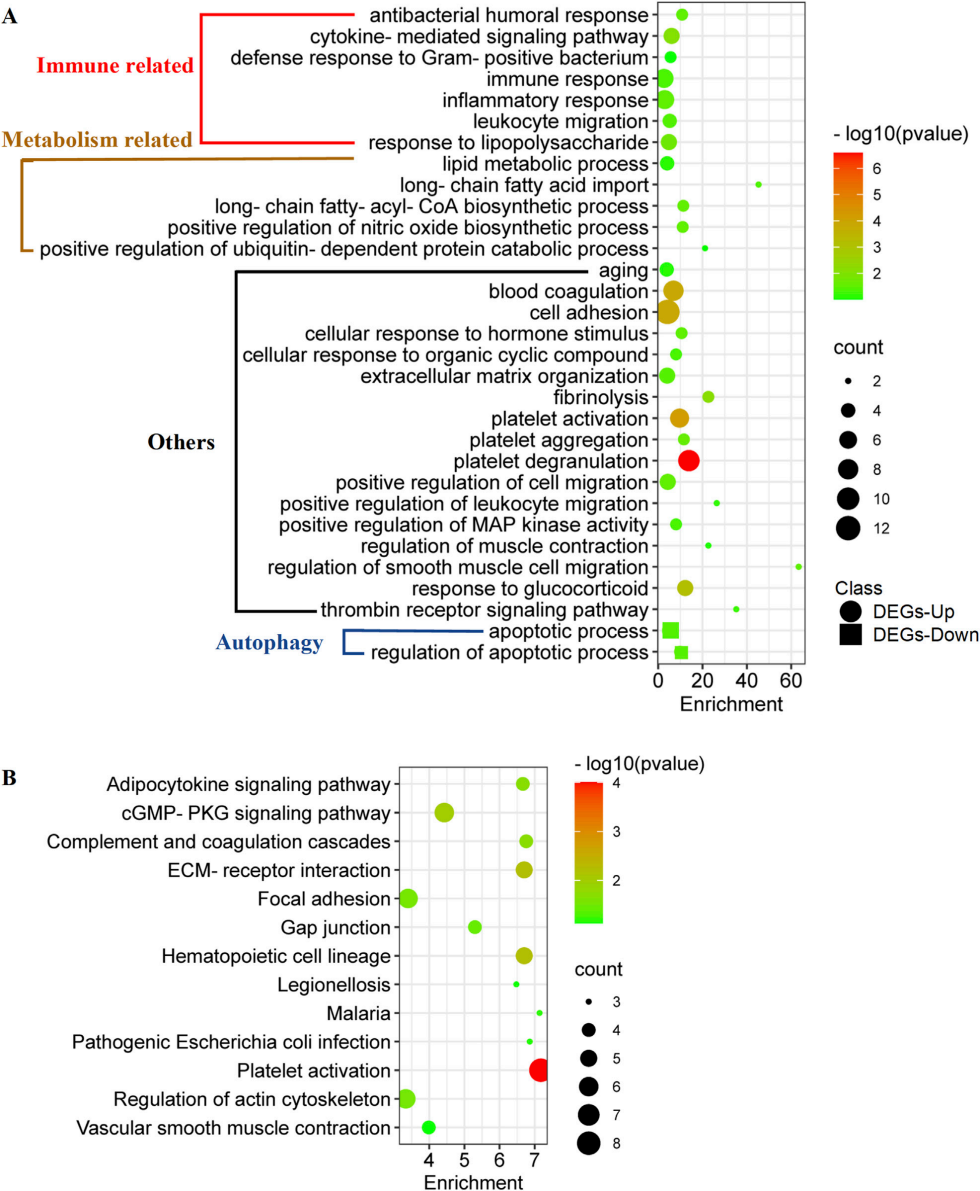
Functional enrichment of co-DEGs in metabolically unhealthy obesity. **(A)** GO analyses of co-DEGs. This analysis clusters the co-DEGs into different biological processes, providing an overview of the functions that these genes are associated with. **(B)** KEGG (Kyoto Encyclopedia of Genes and Genomes) enrichment of co-DEGs.

GO and KEGG analyses were performed to summarize the functional and pathway enrichment of co-DEGs. In GO analyses, 156 co-upregulated genes were enriched in 29 biological process (BP) terms (Figure 2A). Among the BP terms, immune-related biological processes, such as immune response, inflammatory response, and leukocyte migration, and metabolism-related biological processes, such as lipid metabolic process, long-chain fatty-acyl-CoA biosynthetic process, and positive regulation of nitric oxide biosynthetic process, were enriched. In addition, 33 co-downregulated genes were enriched in two BP terms, namely apoptotic process and regulation of apoptotic process (Figure 2A).

Enrichment analysis of the KEGG pathway showed that 156 co-upregulated genes were mainly related to 12 pathways (Figure 2B), such as adipocytokine signaling pathway, cGMP-PKG signaling pathway, and complement and coagulation cascades (Figure 2B). On the other hand, no KEGG pathway was enriched according to the 33 co-downregulated genes.

### Gene interaction network analyses of co-DEGs of MUO

**FIGURE 3:**
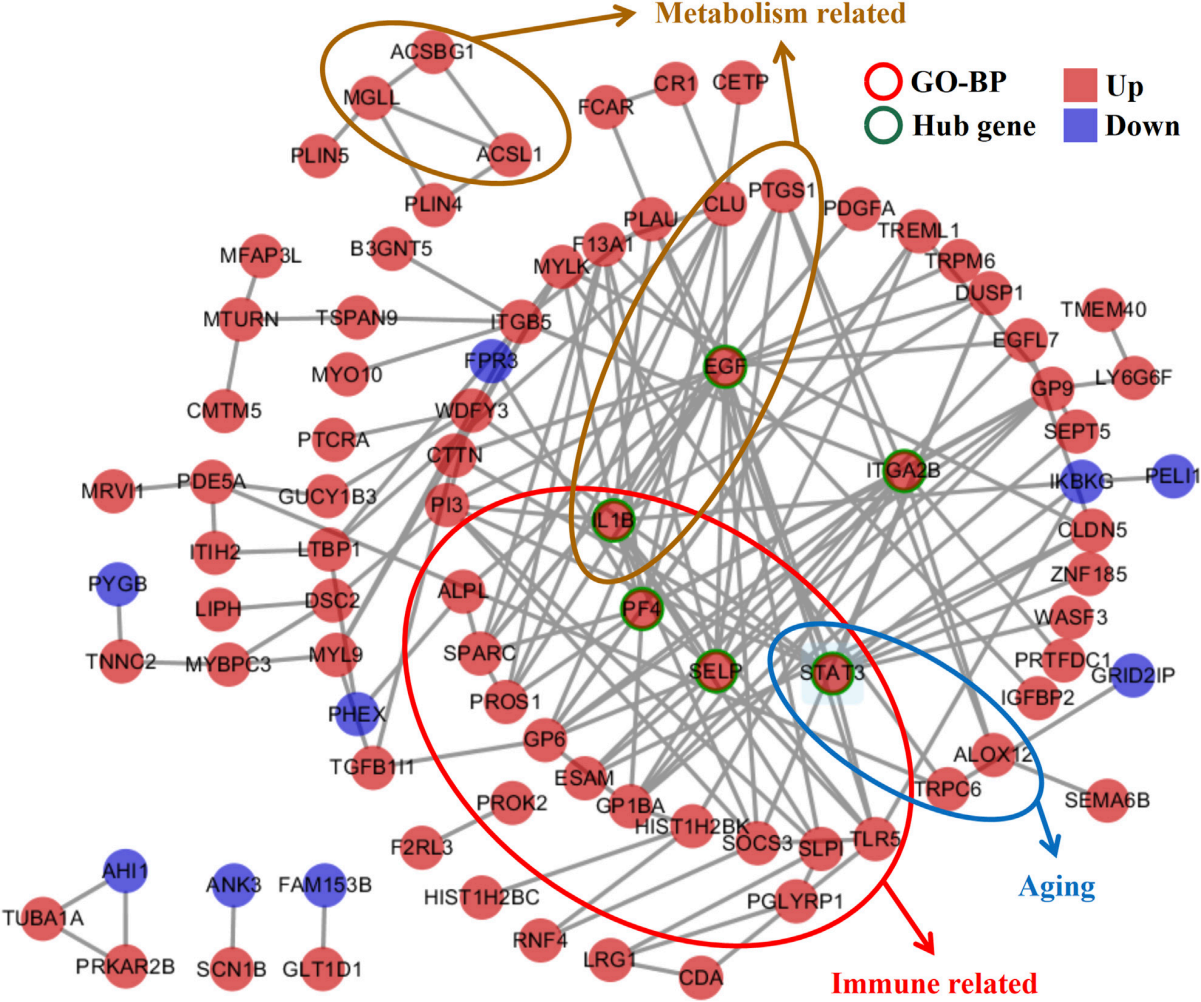
Gene interaction network analyses of the co-DEGs in metabolically unhealthy obesity. The network is composed of nodes (genes) and edges (interactions between genes). The identified hub genes, EGF, STAT3, IL1B, PF4, SELP, and ITGA2B, which interact with at least 10 co-DEGs, are highlighted. Additionally, genes related to immune response, metabolism, and aging are also presented, suggesting their role in regulating the abnormal biological processes in MUO. BP, biological process.

In the interaction network analyses, the 189 co-DEGs were clustered into an interaction network complex containing 89 nodes and 159 edges (Figure 3). Epidermal growth factor (EGF), signal transducer and activator of transcription 3 (STAT3), interleukin 1 beta (IL-1B), platelet factor 4 (PF4), selectin P (SELP), and integrin subunit alpha 2b (ITGA-2B), respectively, interacted with at least ten co-DEGs, which were defined as the hub genes. In addition, immune-, metabolism-, and aging-related genes are presented in Figure 3, which might regulate abnormal biological processes in MUO.

### DEMs and target DEGs in MUO

**FIGURE 4:**
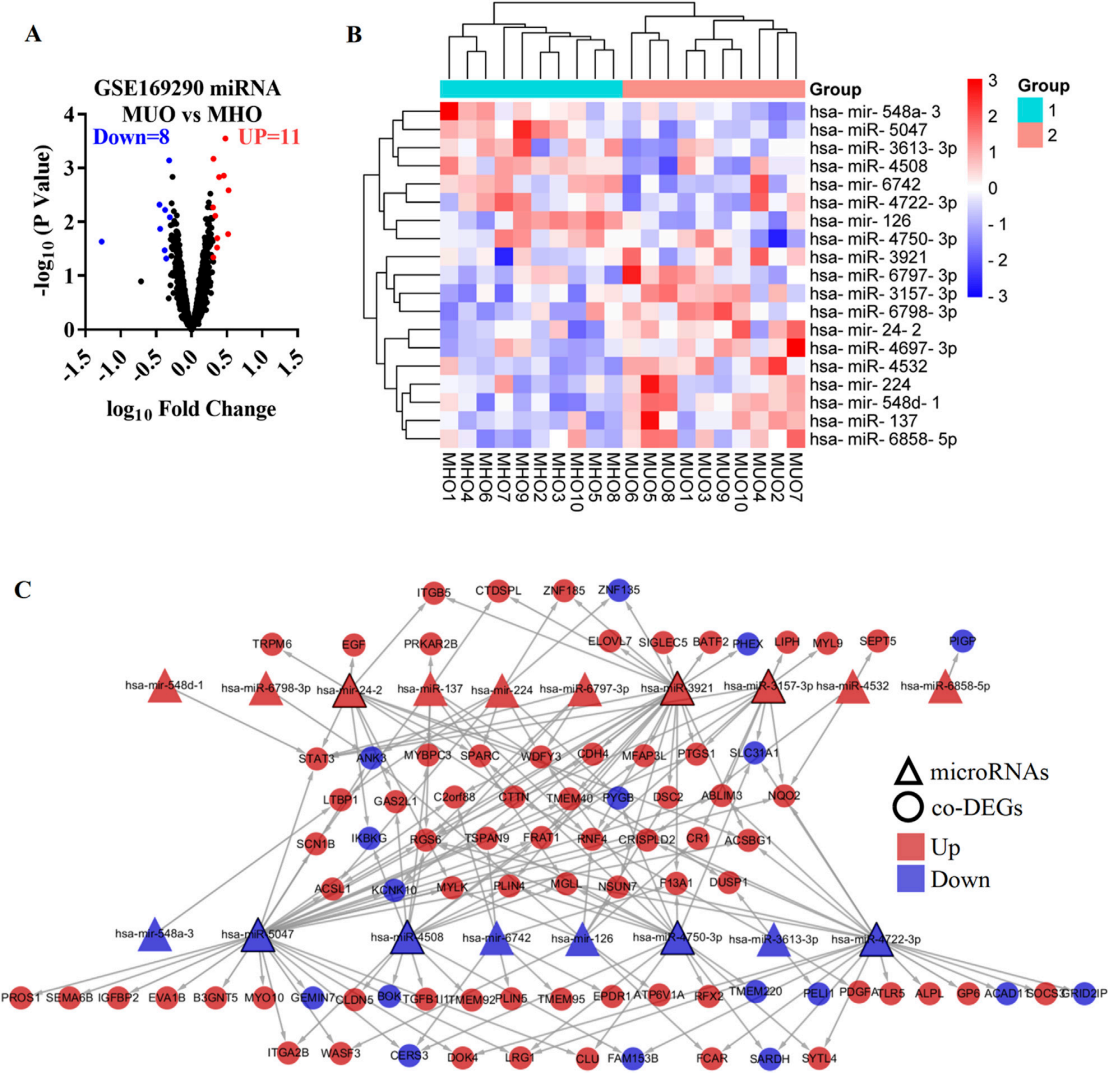
Differentially expressed microRNA targets DEGs in metabolically unhealthy obesity. **(A)** Volcano plot of differentially expressed microRNAs in the MUO group compared with the MHO group, based on the GSE169290 dataset. **(B)** Heatmap of the differentially expressed microRNAs. **(C)** Interaction network of the differentially expressed microRNA and target DEGs in metabolic syndrome. MUO, metabolically unhealthy obesity. MHO, metabolically healthy and obese. DEGs, differentially expressed genes.

The GSE169290 dataset included 10 MUO patients and 10 MHO individuals. Compared with the MHO group, 19 DEMs were identified in the blood of the MUO group, including 11 upregulated microRNAs and eight downregulated microRNAs (Figure 4A). has-miR-137, has-mir-224, has-mir-24-2, has-miR-3157-3p, has-miR-3921, has-miR-4532, has-miR-4697-3p, has-mir-548d-1, has-miR-6797-3p, has-miR-6798-3p, and has-miR-6858-5p were increased in MUO. On the other hand, has-mir-126, has-miR-3613-3p, has-miR-4508, has-miR-4722-3p, has-miR-4750-3p, has-miR-5047, has-mir-548a-3, and has-mir-6742 were decreased in MUO (Figure 4B, Supplementary Table S1).

To predict the target genes of the DEMs, miRNet 2.0, TransmiR v2.0, RNA22, TargetScan 7.2, miRDB, and miRWalk databases were used in the present study (Supplementary Figure S1). Target genes were defined as those predicted by at least three databases. In addition, 9 co-DEGs were predicted as target genes of has-mir-126 (Supplementary Figure S1A), 8 co-DEGs were predicted as target genes of has-miR-137 (Supplementary Figure S1B), 8 co-DEGs were predicted as target genes of has-mir-224 (Supplementary Figure S1C), 11 co-DEGs were predicted as target genes of has-mir-24-2 (Supplementary Figure S1D), 11 co-DEGs were predicted as target genes of has-miR-3157-3p (Supplementary Figure S1E), 4 co-DEGs were predicted as target genes of has-miR-3613-3p (Supplementary Figure S1F), 25 co-DEGs were predicted as target genes of has-miR-3921 (Supplementary Figure S1G), 15 co-DEGs were predicted as target genes of has-miR-4508 (Supplementary Figure S1H), 3 co-DEGs were predicted as target genes of has-miR-4532 (Supplementary Figure S1I), no co-DEGs were predicted as target genes of has-miR-4697-3p (Supplementary Figure S1J), 20 co-DEGs were predicted as target genes of has-miR-4722-3p (Supplementary Figure S1K), 16 co-DEGs were predicted as target genes of has-miR-4750-3p (Supplementary Figure S1L), 30 co-DEGs were predicted as target genes of has-miR-5047 (Supplementary Figure S1M), 1 co-DEG was predicted as the target gene of has-mir-548a-3 (Supplementary Figure S1N), 1 co-DEG was predicted as the target gene of has-mir-548d-1 (Supplementary Figure S1O), 7 co-DEGs were predicted as target genes of has-mir-6742 (Supplementary Figure S1P), 4 co-DEGs were predicted as target genes of has-miR-6797-3p (Supplementary Figure S1Q), 1 co-DEG was predicted as the target gene of has-miR-6798-3p (Supplementary Figure S1R), and 1 co-DEG was predicted as the target gene of has-miR-6858-5p (Supplementary Figure S1S). The DEMs and target genes were present in an interaction network containing 18 microRNA nodes, 85 gene nodes, and 175 edges (Figure 4C). In the network, has-miR-5047, has-miR-3921, has-miR-4722-3p, has-miR-4750-3p, has-miR-4508, has-mir-24-2, and has-miR-3157-3p, respectively, interacted with at least 11 co-DEGs, which were defined as the hub micro-RNA.

## Discussion

In this study, we identified a total of 189 co-differentially expressed genes (co-DEGs) across at least two of the GSE146869, GSE145412, and GSE23561 datasets. These co-DEGs comprised 156 co-upregulated genes and 33 co-downregulated genes. The 156 co-upregulated genes were enriched in 29 BP terms and 12 KEGG pathways. Significantly, the BP terms included enrichments in immune- and metabolism-related processes, highlighting the complex interplay between these two aspects in MUO. In contrast, the 33 co-downregulated genes were only enriched in two BP terms related to the apoptotic process, with no significant KEGG pathway enrichment.

Within the gene interaction network, six hub genes, namely, EGF, STAT3, IL1B, PF4, SELP, and ITGA2B, were identified. These genes likely play crucial roles in the abnormal biological processes associated with MUO. EGF has been linked to higher serum concentrations in patients with metabolic syndrome or obesity (Kim et al., 2020), indicating its potential as a biomarker. The inhibition of the STAT3 signaling pathway has shown promise in reducing visceral obesity (Su et al., 2020), countering metabolic syndrome development (Hua et al., 2018), and preventing obesity-induced neuroinflammation (Zhou et al., 2020). IL1B, a key inflammatory regulator, has been associated with cardiovascular risks induced by obesity and is considered a targeted therapy for metabolic syndrome (Maedler et al., 2011; Amaral et al., 2020). SELP has also been proposed as a target therapy for metabolic syndrome, while increased levels of integrin beta 2 in adipose tissue and blood have been observed in diet-induced obesity (Patel et al., 2017; Mesgari-Abbasi et al., 2021; Wu et al., 2010). Although PF4 may not have a direct reported association with MUO, platelet activation and PF4 secretion are present in obesity (Karamouzis et al., 2011). Therefore, EGF, STAT3, IL1B, SELP, ITGA2B, and PF4 could be markers of MUO and target therapy for the prevention and reversal factors of the transition from MHO to MUO.

We also identified 19 DEMs in the blood of the MUO group using the GSE169290 dataset. Among these, 18 microRNAs targeted 85 genes, which may act as a risk factor for MUO. The potential functions of these DEMs are summarized in Supplementary Table S1. Among the 19 DEMs, three decreased microRNAs (has-mir-126, has-miR-4508, and has-miR-5047) and six increased microRNAs (has-miR-3157-3p, has-miR-137, has-miR-4532, has-miR-4697-3p, has-mir-548d-1, and has-miR-6798-3p) were reported in the blood of MUO (Rovira-Llopis et al., 2021). Thus, the DEMs could be markers of MUO and target therapy for the prevention and reversal factors of the transition from MHO to MUO. Most of these DEMs (has-mir-126 (Park et al., 2018; Yu et al., 2013), has-miR-137 (Deng et al., 2016; Liu et al., 2017), has-miR-3613-3p (Yan et al., 2018; Zhang et al., 2017), has-mir-24-2 (Tang et al., 2019; Zhou et al., 2018), and has-mir-224 (Manzanarez-Ozuna et al., 2018; Kandhavelu et al., 2019; Wu et al., 2013)) were potential biomarkers or potential therapeutic targets for multiple tumors. Interestingly, three DEMs (has-miR-3157-3p (Pala and Denkceken, 2020), has-miR-137 (Li et al., 2017; Jiang et al., 2018), and has-miR-4532 (Li et al., 2021)) have been associated with neurodegenerative diseases, with an increased risk observed in obese patients (Dye et al., 2017). Some of these DEMs have been previously reported in the blood of MUO patients, suggesting their potential as markers and therapeutic targets for preventing and reversing the transition from MHO to MUO. Intriguingly, many of these DEMs are also associated with multiple tumors, and some are linked to neurodegenerative diseases, which are more prevalent in obese patients. However, the functions of five of these DEMs remain unclear, warranting further investigation in animal models or cell-based studies.

Compared to previous research, our study integrated data from multiple datasets in the GEO database, thereby increasing the sample size and enhancing the accuracy of our results (Plaza-Florido et al., 2021; Paczkowska-Abdulsalam et al., 2020; Grayson et al., 2011). Similar to other studies, we found that immune and metabolic functions are intricately involved in the development of MUO (Plaza-Florido et al., 2021; Pandolfi et al., 2016). The co-upregulated genes were enriched in immune- and metabolism-related biological processes, while the co-downregulated genes were associated with the apoptotic process. Additionally, the decreased expression of genes related to autophagy in MUO compared to MHO suggests that promoting protective autophagy, as observed with caloric restriction and weight loss, may be a strategy to prevent or reverse the transition from MHO to MUO (Morselli et al., 2010; Hansen et al., 2008; Szafranski and Mekhail, 2014).

This study innovatively integrates data from multiple GEO datasets. The multi-dataset strategy, distinct from prior limited sample size studies, expands the sample pool. By independently analyzing each dataset and identifying co-DEGs present in at least two datasets, dataset-specific biases are minimized, ensuring that only robustly differentially expressed genes are retained, thus enhancing accuracy. Moreover, while similar studies recognized the role of immune and metabolic functions in MUO, this research uncovers 189 co-DEGs, regulating immune, metabolic, and autophagy-related processes. Identifying 19 DEMs in MUO blood, especially 18 targeting 85 co-DEGs, reveals novel molecular mechanisms, offering potential for more targeted MUO prevention and treatment strategies.

Our findings provide comprehensive insights into the molecular mechanisms underlying MUO. The identified co-DEGs and DEMs, along with their associated pathways, offer potential biomarkers and therapeutic targets. The 189 co-DEGs show promise as therapeutic targets for MUO in several respects. First, modulating key biological processes is a viable approach. The 156 co-upregulated genes enriched in immune- and metabolism- related processes can be targeted to alleviate chronic inflammation and correct abnormal fat metabolism. The 33 co-downregulated genes associated with apoptosis may restore normal cell-death regulation. Second, targeting six identified hub genes can influence abnormal processes. Third, regulating the miRNA–gene interaction network can adjust gene expression and rectify molecular imbalances in MUO. However, further research is needed to fully understand the complex relationships between these molecular components and translate these findings into clinical applications.

## Conclusion

A total of 189 co-DEGs, among which 156 were co-upregulated and 33 were co-downregulated, hold the potential to be biomarkers or therapeutic targets for MUO. These co-DEGs play regulatory roles in immune-, metabolism-, and autophagy-related biological processes within the context of MUO. In addition, in the blood of the MUO group, 19 DEMs were identified. Of these, 18 microRNAs target 85 co-DEGs, which may represent a potential molecular mechanism underlying the occurrence and development of MUO. This finding implies that the intricate interplay between these microRNAs and their target genes could be pivotal in understanding the pathophysiology of MUO, potentially paving the way for more targeted preventive and therapeutic strategies.

## Data Availability

The original contributions presented in the study are included in the article/Supplementary Material, further inquiries can be directed to the corresponding authors.
