# Supplementary material for: Identification of potential crucial genes and mechanisms associated with metabolically unhealthy obesity based on the gene expression profile
Source: Front Genet. 2025 May 1;16:1540721. doi: 10.3389/fgene.2025.1540721 (PMC12078199; doi:10.3389/fgene.2025.1540721)
Supplement: Supplementary file 1 [file DataSheet1.pdf]

Fig. S1

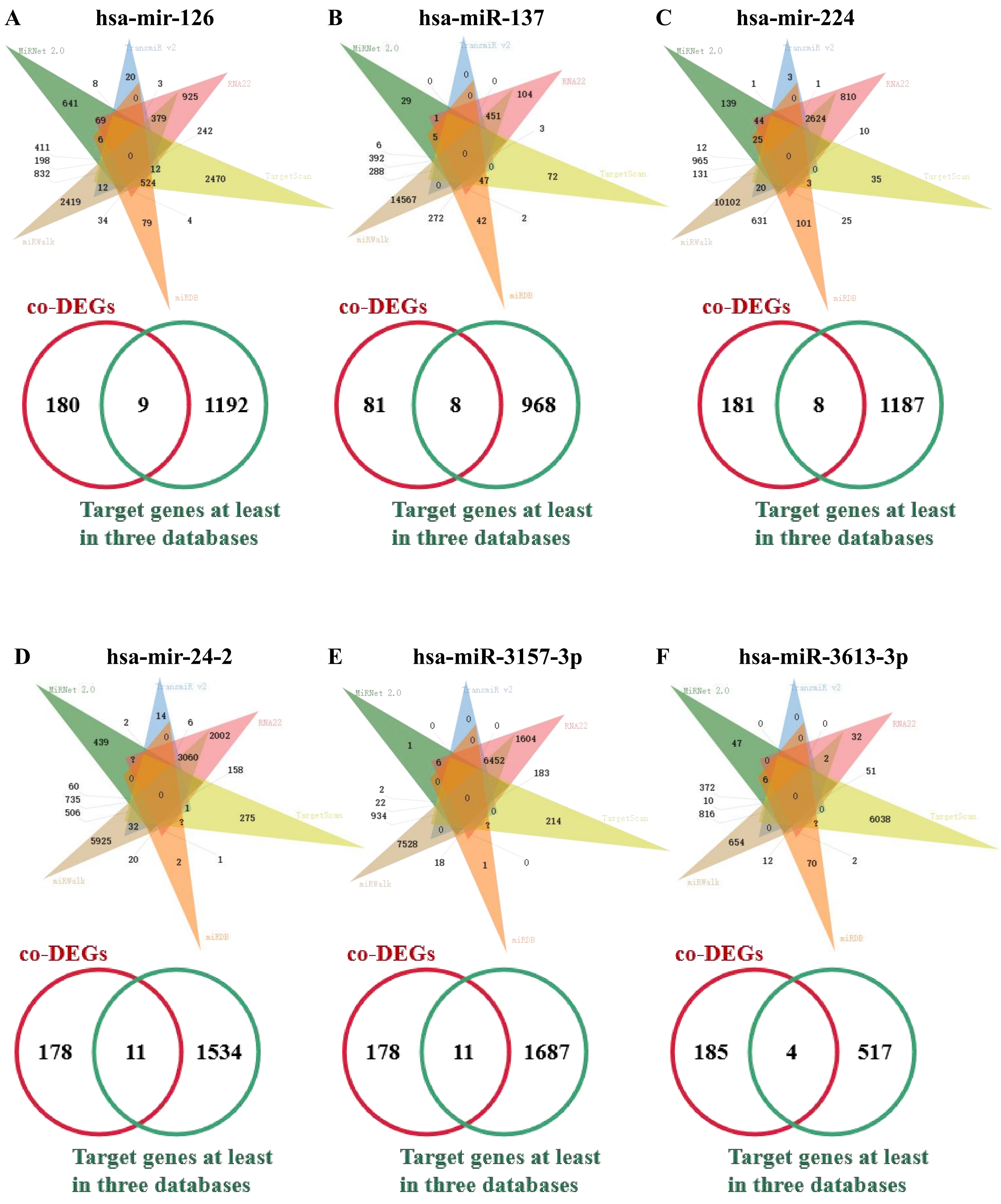

(continued on next page)

Fig. S1 (continued)

G hsa-miR-3921

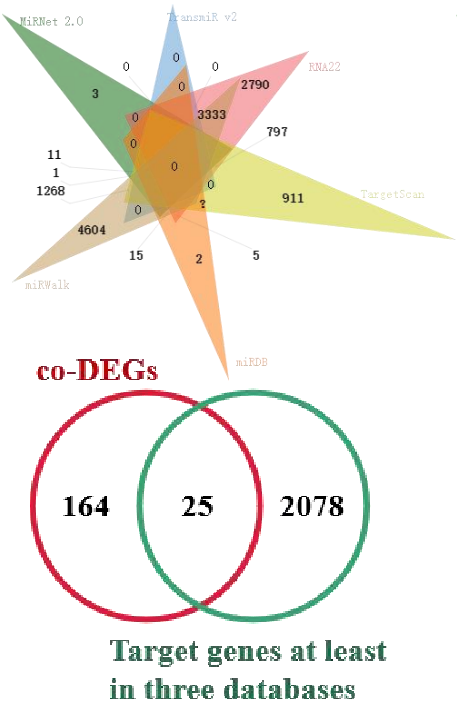

H hsa-miR-4508

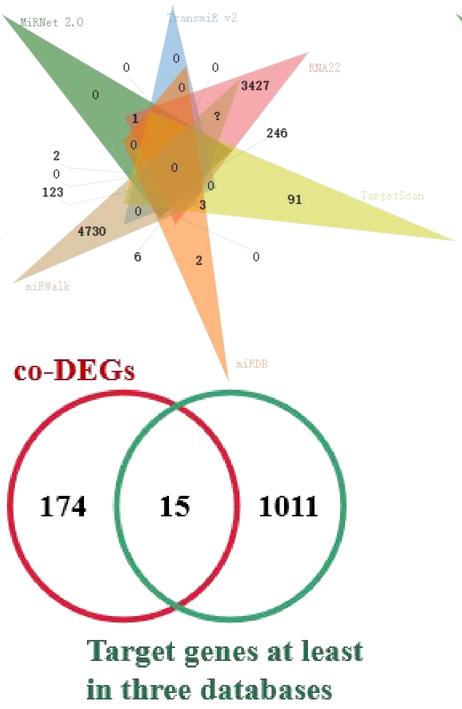

I hsa-miR-4532

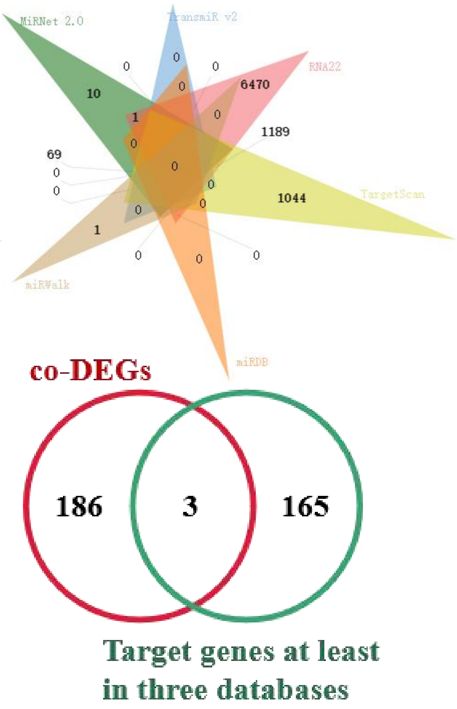

J hsa-miR-4697-3p

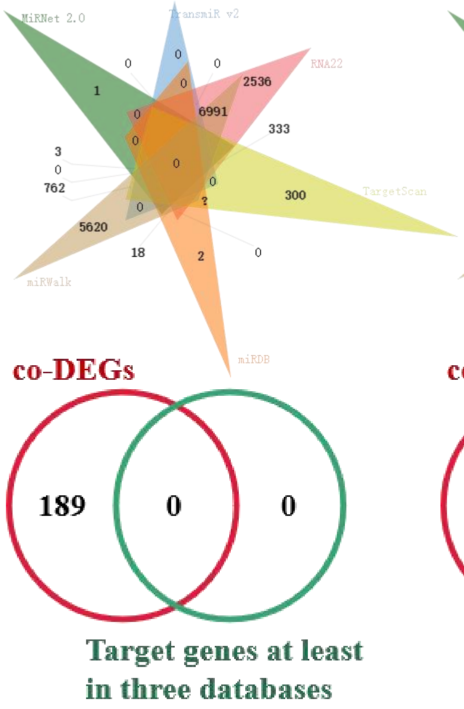

K hsa-miR-4722-3p

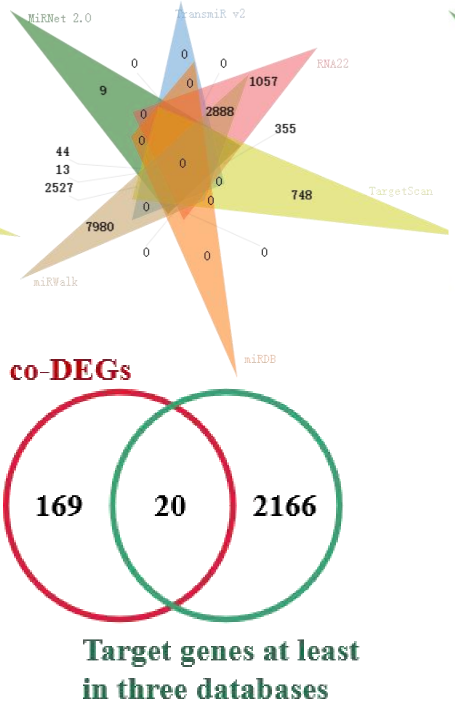

L hsa-miR-4750-3p

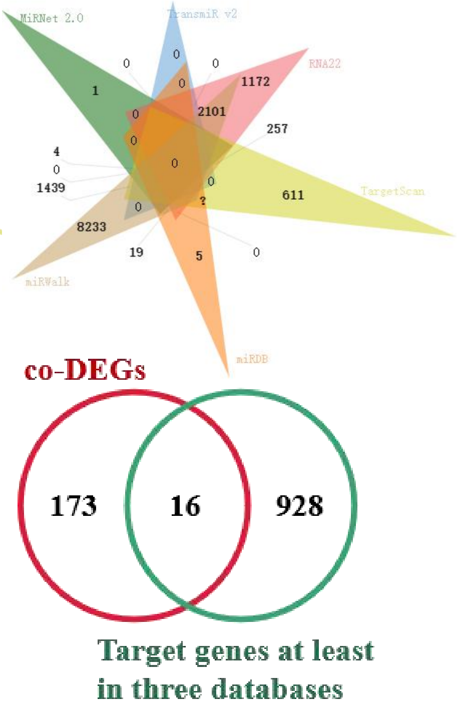

(continued on next page)

Fig. S1 (continued)

M hsa-miR-5047

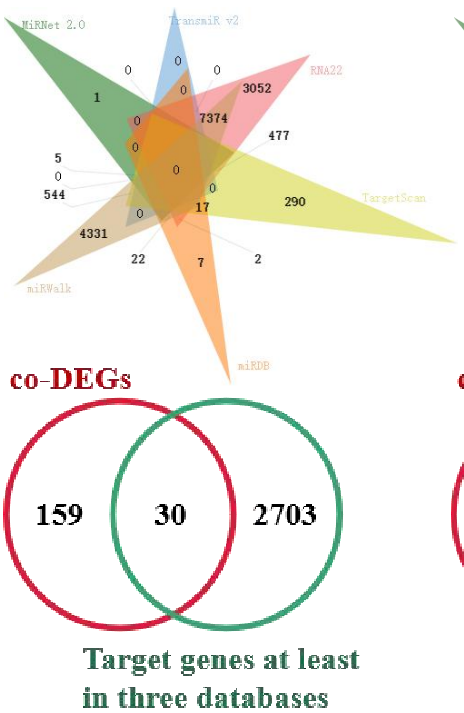

N hsa-mir-548a-3

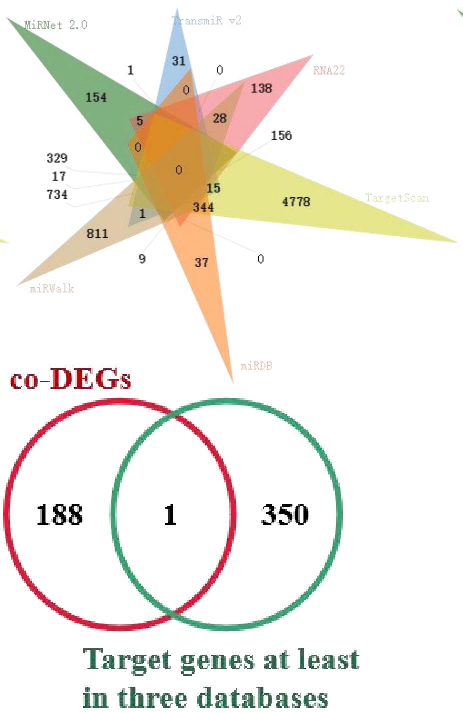

O hsa-mir-548d-1

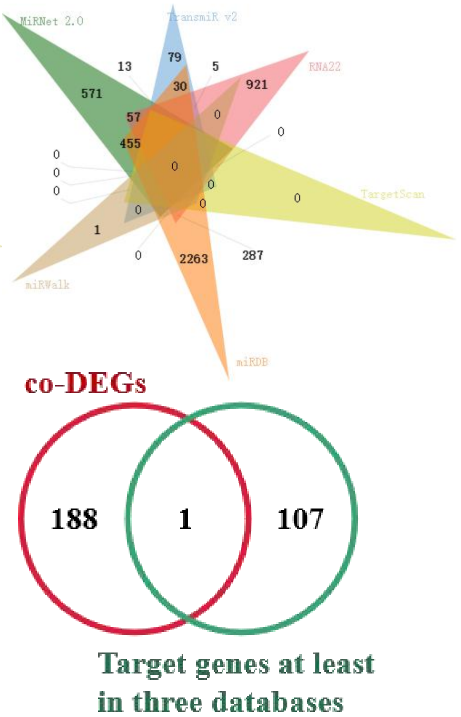

P hsa-mir-6742

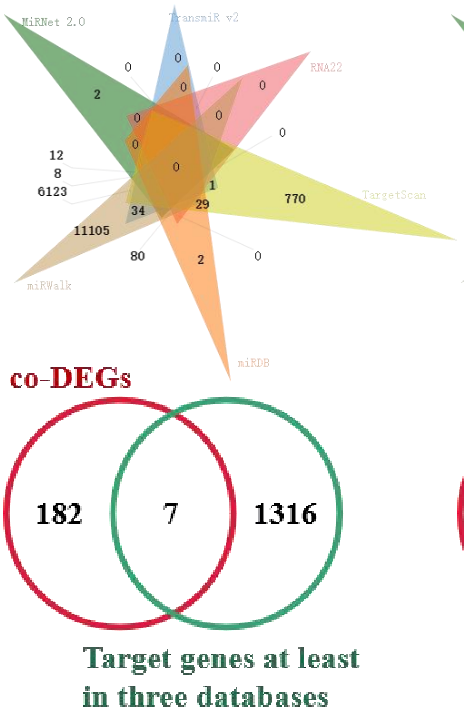

Q hsa-miR-6797-3p

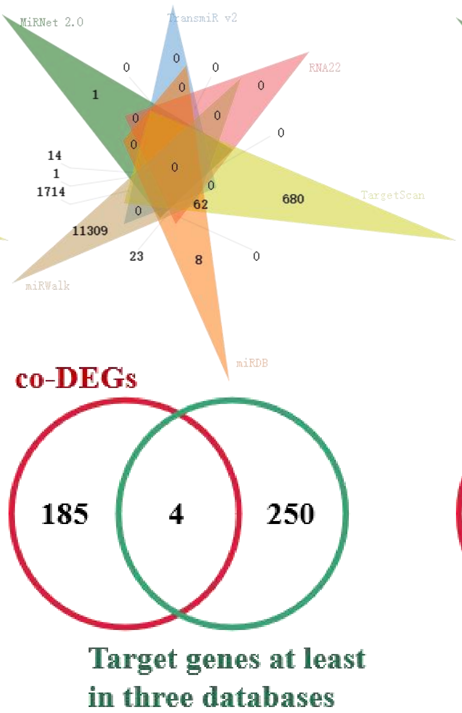

R hsa-miR-6798-3p

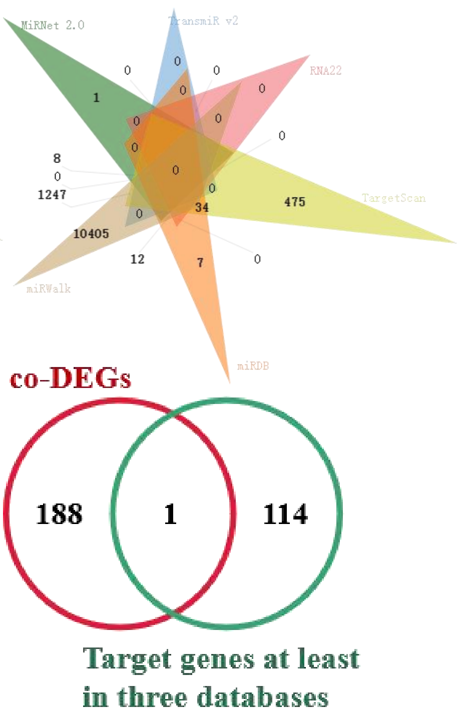

(continued on next page)

Fig. S1 (continued)

S **hsa-miR-6858-5p**

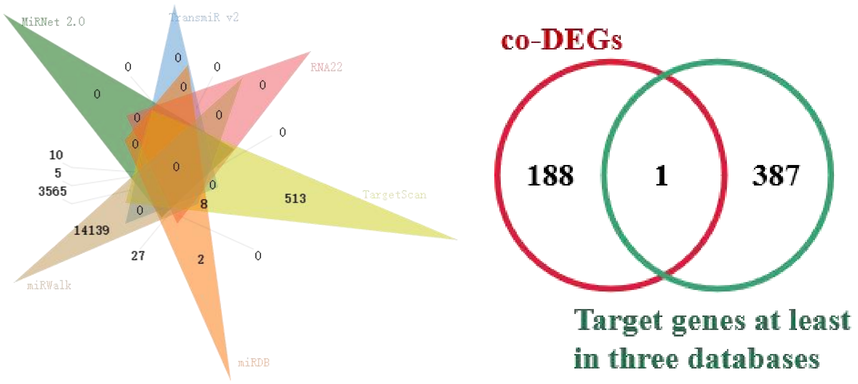

Figure S1. The target genes of differentially expressed microRNAs, and Veen diagram of target genes and co-DEGs in metabolically unhealthy obesity. (A) The target genes of hsa-mir-126 predicted by MiRNet 2.0, TransmiR v2.0, RNA22, TargetScan 7.2, miRDB and miRWalk (up). The Veen diagram of co-DEGs and target genes of hsa-mir-126 (down). (B) The target genes of hsa-miR-137 predicted by MiRNet 2.0, TransmiR v2.0, RNA22, TargetScan 7.2, miRDB and miRWalk (up). The Veen diagram of co-DEGs and target genes of hsa-miR-137 (down). (C) The target genes of hsa-mir-224 predicted by MiRNet 2.0, TransmiR v2.0, RNA22, TargetScan 7.2, miRDB and miRWalk (up). The Veen diagram of co-DEGs and target genes of hsa-mir-224 (down). (D) The target genes of hsa-mir-24-2 predicted by MiRNet 2.0, TransmiR v2.0, RNA22, TargetScan 7.2, miRDB and miRWalk (up). The Veen diagram of co-DEGs and target genes of hsa-mir-24-2 (down). (E) The target genes of hsa-miR-3157-3p predicted by MiRNet 2.0, TransmiR v2.0, RNA22, TargetScan 7.2, miRDB and miRWalk (up). The Veen diagram of co-DEGs and target genes of hsa-miR-3157-3p (down). (F) The target genes of hsa-miR-3613-3p predicted by MiRNet 2.0, TransmiR v2.0, RNA22, TargetScan 7.2, miRDB and miRWalk (up). The Veen diagram of co-DEGs and target genes of hsa-miR-3613-3p (down). (G) The target genes of hsa-miR-3921 predicted by MiRNet 2.0, TransmiR v2.0, RNA22, TargetScan 7.2, miRDB and miRWalk (up). The Veen diagram of co-DEGs and target genes of hsa-miR-3921 (down). (H) The target genes of hsa-miR-4508 predicted by MiRNet 2.0, TransmiR v2.0, RNA22, TargetScan 7.2, miRDB and miRWalk (up). The Veen diagram of co-DEGs and target genes of hsa-miR-4508 (down). (I) The target genes of hsa-miR-4532 predicted by MiRNet 2.0, TransmiR v2.0, RNA22, TargetScan 7.2, miRDB and miRWalk (up). The Veen diagram of co-DEGs and target genes of hsa-miR-4532 (down). (J) The target genes of hsa-miR-4697-3p predicted by MiRNet 2.0, TransmiR v2.0, RNA22, TargetScan 7.2, miRDB and miRWalk (up). The Veen diagram of co-DEGs and target genes of hsa-miR-4697-3p (down). (K) The target genes of hsa-miR-4722-3p predicted by MiRNet 2.0, TransmiR v2.0, RNA22, TargetScan 7.2, miRDB and miRWalk (up). The Veen diagram of co-DEGs and target genes of hsa-miR-4722-3p (down). (L) The target genes of hsa-miR-4750-3p (up). The Veen diagram of co-DEGs and target genes of hsa-miR-4750-3p (down). (M) The target genes of hsa-miR-5047 (up). The Veen diagram of co-DEGs and target genes of hsa-miR-5047 (down). (N) The target genes of hsa-mir-548a-3 (up). The Veen diagram of co-DEGs and target genes of hsa-mir-548a-3 (down). (O) The target genes of hsa-mir-548d-1 (up). The Veen diagram of co-DEGs and target genes of hsa-mir-548d-1 (down). (P) The target genes of hsa-mir-6742 (up). The Veen diagram of co-DEGs and target genes of hsa-mir-6742 (down). (Q) The target genes of hsa-miR-6797-3p (up). The Veen diagram of co-DEGs and target genes of hsa-miR-6797-3p (down). (R) The target genes of hsa-miR-6798-3p (up). The Veen diagram of co-DEGs and target genes of hsa-miR-6798-3p (down). (S) The target genes of hsa-miR-6858-5p (left). The Veen diagram of co-DEGs and target genes of hsa-miR-6858-5p (right). DEGs: differentially expressed genes.

Supplementary Table 1. Details of differentially expressed microRNAs in blood of the MUO group

miRNAs	MUO vs MHO	Sequence Type	Sequence	Potential functions
hsa-mir-126	Down	stem-loop	CGCUGGCGACGGGACAUUAUUACUUUUGGUACGCGCUGUGACACUUCAAACUCGUACCGUGAGUAAUAAUGCGCCGUCCACGGCA	1. Increased in bromocriptine-resistant prolactinomas [1], in blood of patients with diabetic kidney disease [2] and in exosomal of colorectal cancer [3].<br>2. Decreased in human **obesity** [4], **in blood of metabolically unhealthy obese** [5], in plasma of **type 2 diabetes** [6], in urinary of patients with diabetic kidney disease [7], in non-small cell lung cancer [8-11], pancreatic adenocarcinoma [12, 13], in glioblastoma[14, 15] and in the serum of COVID-19 patients [16].<br>3. Potential biomarkers in Natural Killer Cells of Patients with Chronic Fatigue Syndrome / Myalgic Encephalomyelitis [7, 17] and in peripheral blood mononuclear cell of gestational diabetes mellitus patients [18]<br>4. Suppressed PAK4 expression in ovarian cancer SKOV3 cells [19].<br>5. Down-regulates dengue virus replication in Huh-7 cells [20].

miRNAs	MUO vs MHO	Sequence Type	Sequence	Potential functions
hsa-miR-137	Up	miRNA	UUAUUGCUUAAGAA UACGCGUAG	1. Increased **in blood of metabolically unhealthy obese** [5], in gestational diabetes mellitus [21], hepatocellular carcinoma [22], in ovarian cancer [23, 24], in Parkinson's disease [25] and in colonic tissue of Lynch syndrome [26].<br>2. Decreased in astrocytoma [27], in glioblastoma [28], in oligodendroglial tumors [29], in non-small cell lung cancer [30], in gastrointestinal stromal tumor [31], in colorectal carcinoma [32], in papillary thyroid carcinoma [33], in prolactinomas [34], in pancreatic cancer [35, 36], in acute lymphoblastic leukemia [37], renal cell carcinoma [38, 39], in multiple myeloma [40], in cartilage tissue of osteoarthritis [41], in pediatric high-grade gliomas [42], in schizophrenia [43-45], in multiple sclerosis [46], knee osteoarthritis [47] and during the osteogenic differentiation of human adipose-derived stem cells [48].<br>3. Potential therapeutic target for melanoma [49-51], pituitary adenomas [52], acute myeloid leukemia [53], breast cancer [54-56], endometrial cancer [57], Alzheimer's Disease [58] and hypertrophic scars [59].

miRNAs	MUO vs MHO	Sequence Type	Sequence	Potential functions
hsa-mir-224	Up	stem-loop	GGGCUUUCAAGUCACUAGUGGUUCCGUUUAGUAGAUGAUUGUGCAUUGUUUCAAAAUGGUGCCCUAGUGACUACAAAGCCC	1. Increased in lung adenocarcinoma tissues from patients with lymph node metastasis [60], in plasma of patients with hepatocellular carcinoma [61] and CD4+ cells from asymptomatic patients with systemic lupus erythematosus [62].<br>2. Decreased in in medullary thyroid carcinoma [63], during osteogenesis of unrestricted somatic stem cells (USSC) line 1 [64], during lobular neoplasia progression [65], in neuroblastoma [66] and in nasal mucosa of allergic rhinitis [67].<br>3. Potential biomarkers of breast cancer [68] and colon cancer [69].<br>4. Moderate expression in the inner tumor and tumor front of squamous cell lung carcinoma [70].
hsa-mir-24-2	Up	stem-loop	CUCUGCCUCCCGUGCCUACUGAGCUGAAACACAGUUGGUUUGUGUACACUGGCUCAGUUCAGCAGGAACAGGG	Potential biomarkers of breast cancer [71] and metastasis of cervical squamous cell carcinoma [72].
hsa-miR-3157-3p	Up	miRNA	CUGCCCUAGUCUAGCUGAAGCU	1. Increased **in blood of metabolically unhealthy obese** [5].<br>2. Potential biomarkers of schizophrenia [73].
hsa-miR-3613-3p	Down	miRNA	ACAAAAAAAAAAGCCCAACCCUUC	1. Increased in HEK293 cells expressing H5N1 avian influenza virus non-structural protein 1 [74].<br>2. Decreased in colon carcinoma [75].<br>3. Affects cell proliferation and cell cycle in hepatocellular carcinoma [76].
hsa-miR-3921	Up	miRNA	UCUCUGAGUACCAUAUGCCUUGU	Decreased in urine of current smokers [77].

miRNAs	MUO vs MHO	Sequence Type	Sequence	Potential functions
hsa-miR-4508	Down	miRNA	GCGGGGCUGGGCGC GCG	1. Increased after administration of dexmedetomidine [78].<br>2. Decreased **in blood of metabolically unhealthy obese** [5] and in pediatric patients with β-thalassemia [79].
hsa-miR-4532	Up	miRNA	CCCCGGGGAGCCCG GCG	1. Increased **in blood of metabolically unhealthy obese** [5] and in urine of diabetic nephropathy [80, 81].<br>2. Potential biomarkers of schizophrenia [82].<br>3. Carried by acute myeloid leukemia cells -derived exosomes [83].<br>4. Inhibition of miR-4532 protects HLECs from UVR-induced oxidative injury [84].
hsa-miR-4697-3p	Up	miRNA	UGUCAGUGACUCCU GCCCCUUGGU	Increased **in blood of metabolically unhealthy obese** [5].
hsa-miR-4722-3p	Down	miRNA	ACCUGCCAGCACCU CCCUGCAG	Unknown
hsa-miR-4750-3p	Down	miRNA	CCUGACCCACCCCCU CCCGCAG	Unknown
hsa-miR-5047	Down	miRNA	UUGCAGCUGCGGUU GUAAGGU	Decreased **in blood of metabolically unhealthy obese** [5].
hsa-mir-548a-3	Down	stem-loop	CCUAGAAUGUUAUU AGGUCGGUGCAAAA GUAAUUGCGAGUUU UACCAUUACUUUCA AUGGCAAAACUGGC AAUUACUUUUGCAC CAACGUAAUACUU	Unknown

miRNAs	MUO vs MHO	Sequence Type	Sequence	Potential functions
hsa-mir-548d-1	Up	stem-loop	AAACAAGUUAUAUUAGGUUGGUGCAAAAGUAAUUGUGGUUUUUGCCUGUAAAAGUAAUGGCAAAAACCACAGUUUCUUUUGCACCAGACUAAUAAAG	Increased **in blood of metabolically unhealthy obese** [5].
hsa-mir-6742	Down	stem-loop	GAGGGAGUGGGGUGGGACCCAGCUGUUGGCCAUGGCGACAACACCUGGGUUGUCCCCUCUAG	Increased in CVID patients by Ig infusion [85].
hsa-miR-6797-3p	Up	miRNA	UGCAUGACCCUUCCCUCCCCAC	Unknown
hsa-miR-6798-3p	Up	miRNA	CUACCCCCCAUCCCCCUGUAG	Increased **in blood of metabolically unhealthy obese** [5] and in plasma of patients with venous thrombosis [86].
hsa-miR-6858-5p	Up	miRNA	GUGAGGAGGGGCUGGCAGGGAC	Unknown

References

1. Wu ZB, Li WQ, Lin SJ, et al. MicroRNA expression profile of bromocriptine-resistant prolactinomas. Mol Cell Endocrinol. 2014; 395(1-2):10-8.
2. Marques FZ, Campain AE, Tomaszewski M, et al. Gene expression profiling reveals renin mRNA overexpression in human hypertensive kidneys and a role for microRNAs. Hypertension. 2011; 58(6):1093-8.
3. Ma J, Wang P, Huang L, Qiao J, Li J. Bioinformatic analysis reveals an exosomal miRNA-mRNA network in colorectal cancer. BMC Med Genomics. 2021; 14(1):60.
4. Li J, Zhou C, Li J, et al. Global correlation analysis for microRNA and gene expression profiles in human obesity. Pathol Res Pract. 2015; 211(5):361-8.

5. Rovira-Llopis S, Diaz-Rua R, Grau-Del Valle C, et al. Characterization of Differentially Expressed Circulating miRNAs in Metabolically Healthy versus Unhealthy Obesity. Biomedicines. 2021; 9(3).
6. Zampetaki A, Kiechl S, Drozdov I, et al. Plasma microRNA profiling reveals loss of endothelial miR-126 and other microRNAs in type 2 diabetes. Circ Res. 2010; 107(6):810-7.
7. Park S, Moon S, Lee K, Park IB, Lee DH, Nam S. Urinary and Blood MicroRNA-126 and -770 are Potential Noninvasive Biomarker Candidates for Diabetic Nephropathy: a Meta-Analysis. Cell Physiol Biochem. 2018; 46(4):1331-40.
8. Zhu X, Li H, Long L, et al. miR-126 enhances the sensitivity of non-small cell lung cancer cells to anticancer agents by targeting vascular endothelial growth factor A. Acta Biochim Biophys Sin (Shanghai). 2012; 44(6):519-26.
9. Jiao Z, Yu A, He X, et al. Bioinformatics analysis to determine the prognostic value and prospective pathway signaling of miR-126 in non-small cell lung cancer. Ann Transl Med. 2020; 8(24):1639.
10. Zhou CH, Yang SF, Li PQ. Human lung cancer cell line SPC-A1 contains cells with characteristics of cancer stem cells. Neoplasma. 2012; 59(6):685-92.
11. Zhong M, Ma X, Sun C, Chen L. MicroRNAs reduce tumor growth and contribute to enhance cytotoxicity induced by gefitinib in non-small cell lung cancer. Chem Biol Interact. 2010; 184(3):431-8.
12. Wang ZX, Deng TX, Ma Z. Identification of a 4-miRNA signature as a potential prognostic biomarker for pancreatic adenocarcinoma. J Cell Biochem. 2019; 120(10):16416-26.
13. Chen S, Gao C, Yu T, Qu Y, Xiao GG, Huang Z. Bioinformatics Analysis of a Prognostic miRNA Signature and Potential Key Genes in Pancreatic Cancer. Front Oncol. 2021; 11:641289.
14. Yu J, Cai X, He J, Zhao W, Wang Q, Liu B. Microarray-based analysis of gene regulation by transcription factors and microRNAs in glioma. Neurol Sci. 2013; 34(8):1283-9.
15. Feng J, Kim ST, Liu W, et al. An integrated analysis of germline and somatic, genetic and epigenetic alterations at 9p21.3 in glioblastoma. Cancer. 2012; 118(1):232-40.
16. Keikha R, Hashemi-Shahri SM, Jebali A. The relative expression of miR-31, miR-29, miR-126, and miR-17 and their mRNA targets in the serum of COVID-19 patients with different grades during hospitalization. Eur J Med Res. 2021; 26(1):75.
17. Petty RD, McCarthy NE, Le Dieu R, Kerr JR. MicroRNAs hsa-miR-99b, hsa-miR-330, hsa-miR-126 and hsa-miR-30c: Potential Diagnostic Biomarkers in Natural Killer (NK) Cells of Patients with Chronic Fatigue Syndrome (CFS)/ Myalgic Encephalomyelitis (ME). PLoS One. 2016; 11(3):e0150904.
18. Collares CV, Evangelista AF, Xavier DJ, et al. Identifying common and specific microRNAs expressed in peripheral blood mononuclear cell of type 1, type 2, and gestational diabetes mellitus patients. BMC Res Notes. 2013; 6:491.
19. Luo P, Fei J, Zhou J, Zhang W. microRNA-126 suppresses PAK4 expression in ovarian cancer SKOV3 cells. Oncol Lett. 2015; 9(5):2225-29.
20. Kakumani PK, Medigeshi GR, Kaur I, Malhotra P, Mukherjee SK, Bhatnagar RK. Role of human GRP75 in miRNA mediated regulation of dengue virus replication. Gene. 2016; 586(1):7-11.
21. Peng HY, Li MQ, Li HP. MiR-137 Restricts the Viability and Migration of HTR-8/SVneo Cells by Downregulating FNDC5 in Gestational Diabetes Mellitus. Curr Mol Med. 2019; 19(7):494-505.
22. Huang B, Huang M, Li Q. MiR-137 suppresses migration and invasion by targeting EZH2-STAT3 signaling in human hepatocellular carcinoma. Pathol Res Pract. 2018;

214(12):1980-86.
23. Dong P, Xiong Y, Watari H, et al. MiR-137 and miR-34a directly target Snail and inhibit EMT, invasion and sphere-forming ability of ovarian cancer cells. J Exp Clin Cancer Res. 2016; 35(1):132.
24. Song R, Liu Z, Lu L, Liu F, Zhang B. Long Noncoding RNA SCAMP1 Targets miR-137/CXCL12 Axis to Boost Cell Invasion and Angiogenesis in Ovarian Cancer. DNA Cell Biol. 2020; 39(6):1041-50.
25. Li N, Pan X, Zhang J, et al. Plasma levels of miR-137 and miR-124 are associated with Parkinson's disease but not with Parkinson's disease with depression. Neurol Sci. 2017; 38(5):761-67.
26. Zhou C, Li J, Li J, et al. Hsa-miR-137, hsa-miR-520e and hsa-miR-590-3p perform crucial roles in Lynch syndrome. Oncol Lett. 2016; 12(3):2011-17.
27. Deng D, Xue L, Shao N, et al. miR-137 acts as a tumor suppressor in astrocytoma by targeting RASGRF1. Tumour Biol. 2016; 37(3):3331-40.
28. Sun J, Zheng G, Gu Z, Guo Z. MiR-137 inhibits proliferation and angiogenesis of human glioblastoma cells by targeting EZH2. J Neurooncol. 2015; 122(3):481-9.
29. Li KK, Yang L, Pang JC, et al. MIR-137 suppresses growth and invasion, is downregulated in oligodendroglial tumors and targets CSE1L. Brain Pathol. 2013; 23(4):426-39.
30. Liu X, Chen L, Tian XD, Zhang T. MiR-137 and its target TGFA modulate cell growth and tumorigenesis of non-small cell lung cancer. Eur Rev Med Pharmacol Sci. 2017; 21(3):511-17.
31. Liu S, Cui J, Liao G, et al. MiR-137 regulates epithelial-mesenchymal transition in gastrointestinal stromal tumor. Tumour Biol. 2014; 35(9):9131-8.
32. Fasihi A, B MS, Atashi A, Nasiri S. Introduction of hsa-miR-103a and hsa-miR-1827 and hsa-miR-137 as new regulators of Wnt signaling pathway and their relation to colorectal carcinoma. J Cell Biochem. 2018; 119(7):5104-17.
33. Dong S, Jin M, Li Y, Ren P, Liu J. MiR-137 acts as a tumor suppressor in papillary thyroid carcinoma by targeting CXCL12. Oncol Rep. 2016; 35(4):2151-8.
34. Lei C, Jing G, Jichao W, et al. MiR-137's Tumor Suppression on Prolactinomas by Targeting MITF and Modulating Wnt Signaling Pathway. J Clin Endocrinol Metab. 2019; 104(12):6391-402.
35. Lei S, He Z, Chen T, et al. Long noncoding RNA 00976 promotes pancreatic cancer progression through OTUD7B by sponging miR-137 involving EGFR/MAPK pathway. J Exp Clin Cancer Res. 2019; 38(1):470.
36. Ding F, Zhang S, Gao S, et al. MiR-137 functions as a tumor suppressor in pancreatic cancer by targeting MRGBP. J Cell Biochem. 2018; 119(6):4799-807.
37. Huang Y, Zou Y, Zheng R, Ma X. MiR-137 inhibits cell proliferation in acute lymphoblastic leukemia by targeting JARID1B. Eur J Haematol. 2019; 103(3):215-24.
38. Wang M, Gao H, Qu H, Li J, Liu K, Han Z. MiR-137 suppresses tumor growth and metastasis in clear cell renal cell carcinoma. Pharmacol Rep. 2018; 70(5):963-71.
39. Wang L, Li Q, Ye Z, Qiao B. ZBTB7/miR-137 Autoregulatory Circuit Promotes the Progression of Renal Carcinoma. Oncol Res. 2019; 27(9):1007-14.
40. Yang Y, Li F, Saha MN, Abdi J, Qiu L, Chang H. miR-137 and miR-197 Induce Apoptosis and Suppress Tumorigenicity by Targeting MCL-1 in Multiple Myeloma. Clin Cancer Res. 2015; 21(10):2399-411.
41. Pandey MK, Sung B, Ahn KS, Kunnumakkara AB, Chaturvedi MM, Aggarwal BB. Gambogic acid, a novel ligand for transferrin receptor, potentiates TNF-induced apoptosis through modulation of the nuclear factor-kappaB signaling pathway. Blood. 2007;110(10):3517-3525. Blood. 2013; 121(18):3778.

42. Liang ML, Hsieh TH, Ng KH, et al. Downregulation of miR-137 and miR-6500-3p promotes cell proliferation in pediatric high-grade gliomas. Oncotarget. 2016; 7(15):19723-37.
43. Yin J, Lin J, Luo X, et al. miR-137: a new player in schizophrenia. Int J Mol Sci. 2014; 15(2):3262-71.
44. Pacheco A, Berger R, Freedman R, Law AJ. A VNTR Regulates miR-137 Expression Through Novel Alternative Splicing and Contributes to Risk for Schizophrenia. Sci Rep. 2019; 9(1):11793.
45. Ma J, Shang S, Wang J, et al. Identification of miR-22-3p, miR-92a-3p, and miR-137 in peripheral blood as biomarker for schizophrenia. Psychiatry Res. 2018; 265:70-76.
46. Senousy MA, Shaker OG, Sayed NH, Fathy N, Kortam MA. LncRNA GAS5 and miR-137 Polymorphisms and Expression are Associated with Multiple Sclerosis Risk: Mechanistic Insights and Potential Clinical Impact. ACS Chem Neurosci. 2020; 11(11):1651-60.
47. Gao ST, Yu YM, Wan LP, Liu ZM, Lin JX. LncRNA GAS5 induces chondrocyte apoptosis by down-regulating miR-137. Eur Rev Med Pharmacol Sci. 2020; 24(21):10984-91.
48. Ma X, Fan C, Wang Y, et al. MiR-137 knockdown promotes the osteogenic differentiation of human adipose-derived stem cells via the LSD1/BMP2/SMAD4 signaling network. J Cell Physiol. 2020; 235(2):909-19.
49. Luo M, Wu L, Zhang K, et al. miR-137 regulates ferroptosis by targeting glutamine transporter SLC1A5 in melanoma. Cell Death Differ. 2018; 25(8):1457-72.
50. Sun L, Bian G, Meng Z, Dang G, Shi D, Mi S. MiR-144 Inhibits Uveal Melanoma Cell Proliferation and Invasion by Regulating c-Met Expression. PLoS One. 2015; 10(5):e0124428.
51. Hao S, Luo C, Abukiwan A, et al. miR-137 inhibits proliferation of melanoma cells by targeting PAK2. Exp Dermatol. 2015; 24(12):947-52.
52. Wei D, Yu Z, Cheng Y, et al. Dysregulated miR-137 and its target EGFR contribute to the progression of pituitary adenomas. Mol Cell Endocrinol. 2021; 520:111083.
53. Hu Y, Dong X, Chu G, et al. miR-137 downregulates c-kit expression in acute myeloid leukemia. Leuk Res. 2017; 57:72-77.
54. Du F, Yu L, Wu Y, et al. miR-137 alleviates doxorubicin resistance in breast cancer through inhibition of epithelial-mesenchymal transition by targeting DUSP4. Cell Death Dis. 2019; 10(12):922.
55. Cheng S, Huang Y, Lou C, He Y, Zhang Y, Zhang Q. FSTL1 enhances chemoresistance and maintains stemness in breast cancer cells via integrin beta3/Wnt signaling under miR-137 regulation. Cancer Biol Ther. 2019; 20(3):328-37.
56. Chen F, Luo N, Hu Y, Li X, Zhang K. MiR-137 Suppresses Triple-Negative Breast Cancer Stemness and Tumorigenesis by Perturbing BCL11A-DNMT1 Interaction. Cell Physiol Biochem. 2018; 47(5):2147-58.
57. Zhang W, Chen JH, Shan T, et al. miR-137 is a tumor suppressor in endometrial cancer and is repressed by DNA hypermethylation. Lab Invest. 2018; 98(11):1397-407.
58. Jiang Y, Xu B, Chen J, et al. Micro-RNA-137 Inhibits Tau Hyperphosphorylation in Alzheimer's Disease and Targets the CACNA1C Gene in Transgenic Mice and Human Neuroblastoma SH-SY5Y Cells. Med Sci Monit. 2018; 24:5635-44.
59. Zhang Q, Guo B, Hui Q, Chang P, Tao K. miR-137 Inhibits Proliferation and Metastasis of Hypertrophic Scar Fibroblasts via Targeting Pleiotrophin. Cell Physiol Biochem. 2018; 49(3):985-95.
60. Wang Y, Shang S, Yu K, Sun H, Ma W, Zhao W. miR-224, miR-147b and miR-31 associated with lymph node metastasis and prognosis for lung adenocarcinoma by regulating PRPF4B, WDR82 or NR3C2. PeerJ. 2020; 8:e9704.
61. Amr KS, Elmawgoud Atia HA, Elazeem Elbnhawy RA, Ezzat WM. Early diagnostic evaluation of miR-122 and miR-224 as biomarkers for hepatocellular carcinoma. Genes Dis.

2017; 4(4):215-21.
62. Martinez-Ramos R, Garcia-Lozano JR, Lucena JM, et al. Differential expression pattern of microRNAs in CD4+ and CD19+ cells from asymptomatic patients with systemic lupus erythematosus. Lupus. 2014; 23(4):353-9.
63. Cavedon E, Barollo S, Bertazza L, et al. Prognostic Impact of miR-224 and RAS Mutations in Medullary Thyroid Carcinoma. Int J Endocrinol. 2017; 2017:4915736.
64. Schaap-Oziemlak AM, Raymakers RA, Bergevoet SM, et al. MicroRNA hsa-miR-135b regulates mineralization in osteogenic differentiation of human unrestricted somatic stem cells. Stem Cells Dev. 2010; 19(6):877-85.
65. Giricz O, Reynolds PA, Ramnauth A, et al. Hsa-miR-375 is differentially expressed during breast lobular neoplasia and promotes loss of mammary acinar polarity. J Pathol. 2012; 226(1):108-19.
66. Althoff K, Beckers A, Odersky A, et al. MiR-137 functions as a tumor suppressor in neuroblastoma by downregulating KDM1A. Int J Cancer. 2013; 133(5):1064-73.
67. Shaoqing Y, Ruxin Z, Guojun L, et al. Microarray analysis of differentially expressed microRNAs in allergic rhinitis. Am J Rhinol Allergy. 2011; 25(6):e242-6.
68. Manzanarez-Ozuna E, Flores DL, Gutierrez-Lopez E, Cervantes D, Juarez P. Model based on GA and DNN for prediction of mRNA-Smad7 expression regulated by miRNAs in breast cancer. Theor Biol Med Model. 2018; 15(1):24.
69. Kandhavelu J, Subramanian K, Khan A, Omar A, Ruff P, Penny C. Computational Analysis of miRNA and their Gene Targets Significantly Involved in Colorectal Cancer Progression. Microrna. 2019; 8(1):68-75.
70. Wu H, Haag D, Muley T, et al. Tumor-microenvironment interactions studied by zonal transcriptional profiling of squamous cell lung carcinoma. Genes Chromosomes Cancer. 2013; 52(3):250-64.
71. Tang J, Ma W, Zeng Q, Tan J, Cao K, Luo L. Identification of miRNA-Based Signature as a Novel Potential Prognostic Biomarker in Patients with Breast Cancer. Dis Markers. 2019; 2019:3815952.
72. Zhou J, Liu X, Wang C, Li C. The correlation analysis of miRNAs and target genes in metastasis of cervical squamous cell carcinoma. Epigenomics. 2018; 10(3):259-75.
73. Pala E, Denkceken T. Evaluation of miRNA Expression Profiles in Schizophrenia Using Principal-Component Analysis-Based Unsupervised Feature Extraction Method. J Comput Biol. 2020; 27(8):1253-63.
74. Jiao H, Zheng Z, Shuai X, et al. MicroRNA expression profiles from HEK293 cells expressing H5N1 avian influenza virus non-structural protein 1. Innate Immun. 2019; 25(2):110-17.
75. Yan W, Yang W, Liu Z, Wu G. Characterization of microRNA expression in primary human colon adenocarcinoma cells (SW480) and their lymph node metastatic derivatives (SW620). Onco Targets Ther. 2018; 11:4701-09.
76. Zhang D, Liu E, Kang J, Yang X, Liu H. MiR-3613-3p affects cell proliferation and cell cycle in hepatocellular carcinoma. Oncotarget. 2017; 8(54):93014-28.
77. Navratilova Z, Losse S, Petrova P, Sikorova K, Chabronova A, Petrek M. The Effect of Tobacco Smoking and Smoking Cessation on Urinal miRNAs in a Pilot Study. Life (Basel). 2020; 10(9).
78. Yang X, Chen H, Chen Y, et al. Circulating miRNA Expression Profiling and Target Prediction in Patients Receiving Dexmedetomidine. Cell Physiol Biochem. 2018; 50(2):552-68.

79. Wang H, Chen M, Xu S, et al. Abnormal regulation of microRNAs and related genes in pediatric beta-thalassemia. J Clin Lab Anal. 2021:e23945.
80. Chen ZR, He FZ, Liu MZ, et al. MIR4532 gene variant rs60432575 influences the expression of KCNJ11 and the sulfonylureas-stimulated insulin secretion. Endocrine. 2019; 63(3):489-96.
81. Monteiro MB, Santos-Bezerra DP, Pelaes TS, Vaidya VS, Correa-Giannella ML. MicroRNAs 1915-3p, 2861, and 4532 Are Associated with Long-Term Renal Function Decline in Type 1 Diabetes. Clin Chem. 2019; 65(11):1458-59.
82. Li R, Wang Q, Qiu Y, et al. A Potential Autophagy-Related Competing Endogenous RNA Network and Corresponding Diagnostic Efficacy in Schizophrenia. Front Psychiatry. 2021; 12:628361.
83. Zhao C, Du F, Zhao Y, Wang S, Qi L. Acute myeloid leukemia cells secrete microRNA-4532-containing exosomes to mediate normal hematopoiesis in hematopoietic stem cells by activating the LDOC1-dependent STAT3 signaling pathway. Stem Cell Res Ther. 2019; 10(1):384.
84. Sun GL, Huang D, Li KR, Jiang Q. microRNA-4532 inhibition protects human lens epithelial cells from ultra-violet-induced oxidative injury via activating SIRT6-Nrf2 signaling. Biochem Biophys Res Commun. 2019; 514(3):777-84.
85. De Felice B, Nigro E, Polito R, et al. Differently expressed microRNA in response to the first Ig replacement therapy in common variable immunodeficiency patients. Sci Rep. 2020; 10(1):21482.
86. Gabler J, Basilio J, Steinbrecher O, Kollars M, Kyrle PA, Eichinger S. MicroRNA Signatures in Plasma of Patients With Venous Thrombosis: A Preliminary Report. Am J Med Sci. 2021; 361(4):509-16.
